# Prospects for Malaria Elimination in Mesoamerica and Hispaniola

**DOI:** 10.1371/journal.pntd.0003700

**Published:** 2015-05-14

**Authors:** Sócrates Herrera, Sergio Andrés Ochoa-Orozco, Iveth J. González, Lucrecia Peinado, Martha L. Quiñones, Myriam Arévalo-Herrera

**Affiliations:** 1 Caucaseco Scientific Research Center, Cali, Colombia; 2 Malaria Vaccine and Drug Development Center, Cali, Colombia; 3 Foundation for Innovative New Diagnostics (FIND), Geneva, Switzerland; 4 Health Management Consultant, Guatemala City, Guatemala; 5 Universidad Nacional de Colombia, Bogotá, Colombia; 6 Facultad de Salud, Universidad del Valle, Cali, Colombia; The George Washington University School of Medicine and Health Sciences, UNITED STATES

## Abstract

Malaria remains endemic in 21 countries of the American continent with an estimated 427,000 cases per year. Approximately 10% of these occur in the Mesoamerican and Caribbean regions. During the last decade, malaria transmission in Mesoamerica showed a decrease of ~85%; whereas, in the Caribbean region, Hispaniola (comprising the Dominican Republic [DR] and Haiti) presented an overall rise in malaria transmission, primarily due to a steady increase in Haiti, while DR experienced a significant transmission decrease in this period.

The significant malaria reduction observed recently in the region prompted the launch of an initiative for Malaria Elimination in Mesoamerica and Hispaniola (EMMIE) with the active involvement of the National Malaria Control Programs (NMCPs) of nine countries, the Regional Coordination Mechanism (RCM) for Mesoamerica, and the Council of Health Ministries of Central America and Dominican Republic (COMISCA). The EMMIE initiative is supported by the Global Fund for Aids, Tuberculosis and Malaria (GFATM) with active participation of multiple partners including Ministries of Health, bilateral and multilateral agencies, as well as research centers. EMMIE’s main goal is to achieve elimination of malaria transmission in the region by 2020. Here we discuss the prospects, challenges, and research needs associated with this initiative that, if successful, could represent a paradigm for other malaria-affected regions.

## Introduction

Despite its global decreasing trend, malaria remains an important public health problem worldwide, affecting mainly developing countries in Africa, Asia, and Latin America (LA). After several decades of steady increase, from about 2000 when ~500 million cases were reported, malaria incidence decreased and, in 2013, an estimated ~198 million clinical malaria cases and ~584,000 deaths were reported [1], representing a global morbidity decrease of >50% and mortality of ~45% [1,2]. Approximately 80% of these cases were caused by *Plasmodium falciparum*, followed by ~20% *P*. *vivax* infections, with a limited number of cases caused by *P*. *malariae*, *P*. *ovale*, and *P*. *knowlesi* [1].

Although malaria burden remains high, this significant reduction over 12 years is encouraging. The “Millennium Development Goal 6” (MDG 6) and the Roll Back Malaria (RBM) initiatives, which monitored the progress on malaria reduction, proposed to reverse the malaria incidence trend by 2015 and reduce the number of clinical cases reported by 75% in 2000. Currently, 59 of 103 countries with confirmed malaria transmission in 2000 have achieved the MDG 6 objective, and 52 others are progressing towards this goal [3]. It is remarkable that 20 countries are on track to eliminate malaria, ten are in the pre-elimination phase, five are in elimination phase, and another five are in re-introduction prevention phases [1,4]. Although numerous American countries still have endemic transmission of malaria, they account for only 1.0% of the global malaria cases, with several countries fulfilling conditions to embark on malaria-elimination efforts and several others already in pre-elimination [1,5].

## Malaria Epidemiology in the American Continent

**Fig 1:**
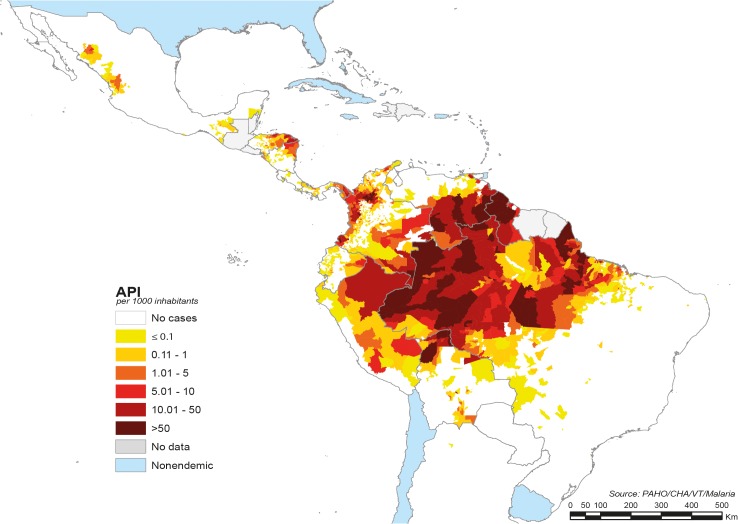
Distribution of malaria in the American continent according to the Annual Parasite Index in 2012. Source: Pan American Health Organization (PAHO) (No data available from Guatemala and El Salvador).

Approximately 120 million people in 21 American countries live at risk of malaria infection within the context of hypoendemic and unstable transmission [1,6,7]. The Amazon basin shows the greatest transmission involving five countries with ~90% of the malaria burden in the continent (Fig 1). *P*. *vivax* accounts for ~74% of the cases, *P*. *falciparum* for ~26%, and a limited number of cases (~0.1%) are caused by *P*. *malariae* [5].

After decades of steady malaria increase with transmission spikes in different countries, in 1994, LA presented an overall peak of ~1.2 million cases, after which this trend was reversed [8]. Malaria kept decreasing as a result of increased local investment in control and surveillance activities, as well as significant external financial and technical support. The RBM initiative was adopted in the Americas in 2000 [9] after which the Pan American Health Organization (PAHO) established a Regional Strategy for Malaria in the Americas (2006–2010), a multi-stakeholder effort that significantly contributed to accelerate this malaria decrease [10] and, more recently, a Strategy and Plan of Action for Malaria (2011–2015) [11]. Meanwhile, 15 countries received substantial financial support from the GFATM, and the entire region benefited from the Amazon Malaria Initiative (AMI) sponsored mainly by the U.S. Agency for International Development (USAID), and the active participation of PAHO, the Centers for Disease Prevention and Control (CDC), and the U.S. Pharmacopeia [12]. Since 1994, disease burden in the region has decreased to ~469,000 cases [1], 15 countries have reached the RBM goal of 75% reduction in 2000, and Peru appears to be able to achieve it in 2015. Furthermore, Argentina, Belize, Costa Rica, El Salvador, Ecuador, Mexico, and Paraguay are in the pre-elimination phase [3,13]. Based on malaria epidemiology and development, the continent can be divided into three regions: South America (SA), including the Amazon basin and the Andean region; Mesoamerica; and the Caribbean islands.

## Malaria in South America

Despite substantial malaria increase in SA between the 1960s and 1990s due to multiple factors, including parasite resistance to chloroquine and sulfadoxine-pyrimethamine [12,14,15], appearance and spreading of DDT resistance [16,17], decentralization of health systems with integration of the vertical malaria control programs, and reduction of resources [8], in the 2000–2012 period, malaria transmission in SA experienced a great overall reduction (80.3%). There were periodic epidemic spikes associated to climate changes caused by “El Niño” Southern Oscillation (ENSO), which mainly affected Colombia, Ecuador, and Peru [13,18], but also countries like Venezuela and Guyana [18–20]. Almost 52% of the malaria cases in the continent are from Brazil, followed by Colombia (12.8%), Venezuela (11.2%), Peru (6.7%), and Guyana (6.7%) [5], indicating that ~90% of the SA malaria cases are from the Amazon basin [13,21] (Fig 1). Guyana and Venezuela are the only countries with recent malaria increases [5,22], with an increasing proportion of *P*. *falciparum* cases. Countries such as Surinam and French Guiana have experienced expansion of gold mining accompanied by population migration [23–25]. The remaining 10% is being transmitted in non-Amazonian regions: Andean regions with areas of Peru, Ecuador, and Colombia showing less transmission intensity, mainly in lowlands near the Pacific coast. A project sponsored by the GFTAM to reinforce malaria control activities at countries’ borders (PAMAFRO) [26] significantly reduced the disease burden in these regions generating a positive scenario for elimination efforts. However, the northwestern region of Colombia, in proximity to Panama, is among the most endemic of this country representing a threat for EMMIE.

## Malaria in Mesoamerica

**Fig 2:**
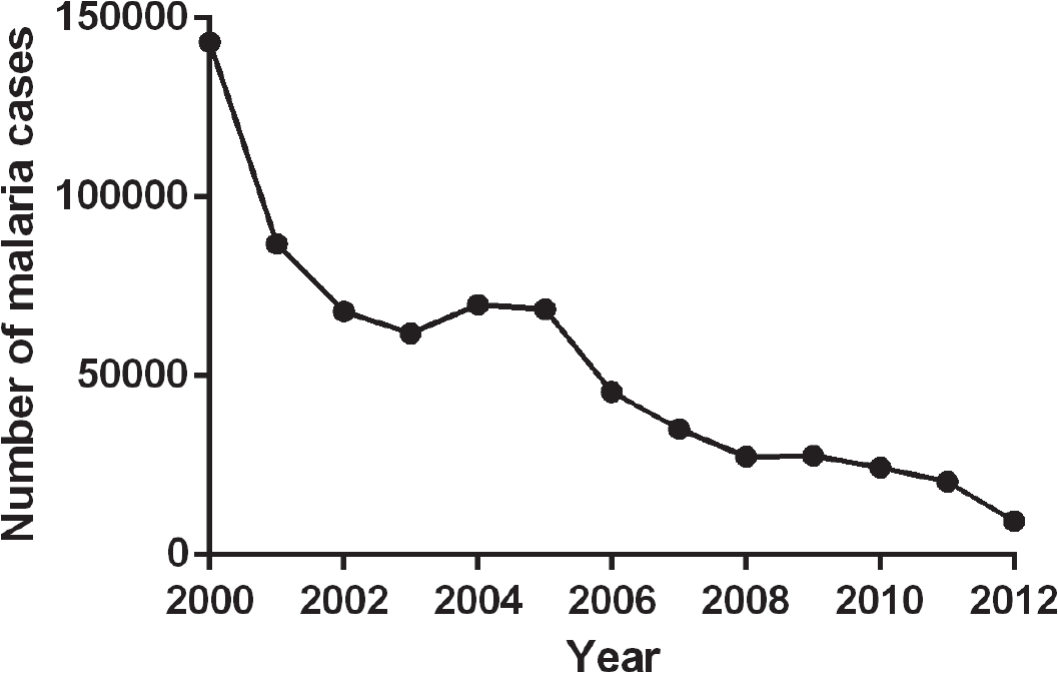
Malaria cases in Mesoamerica from 2000 to 2012.

**Fig 3:**
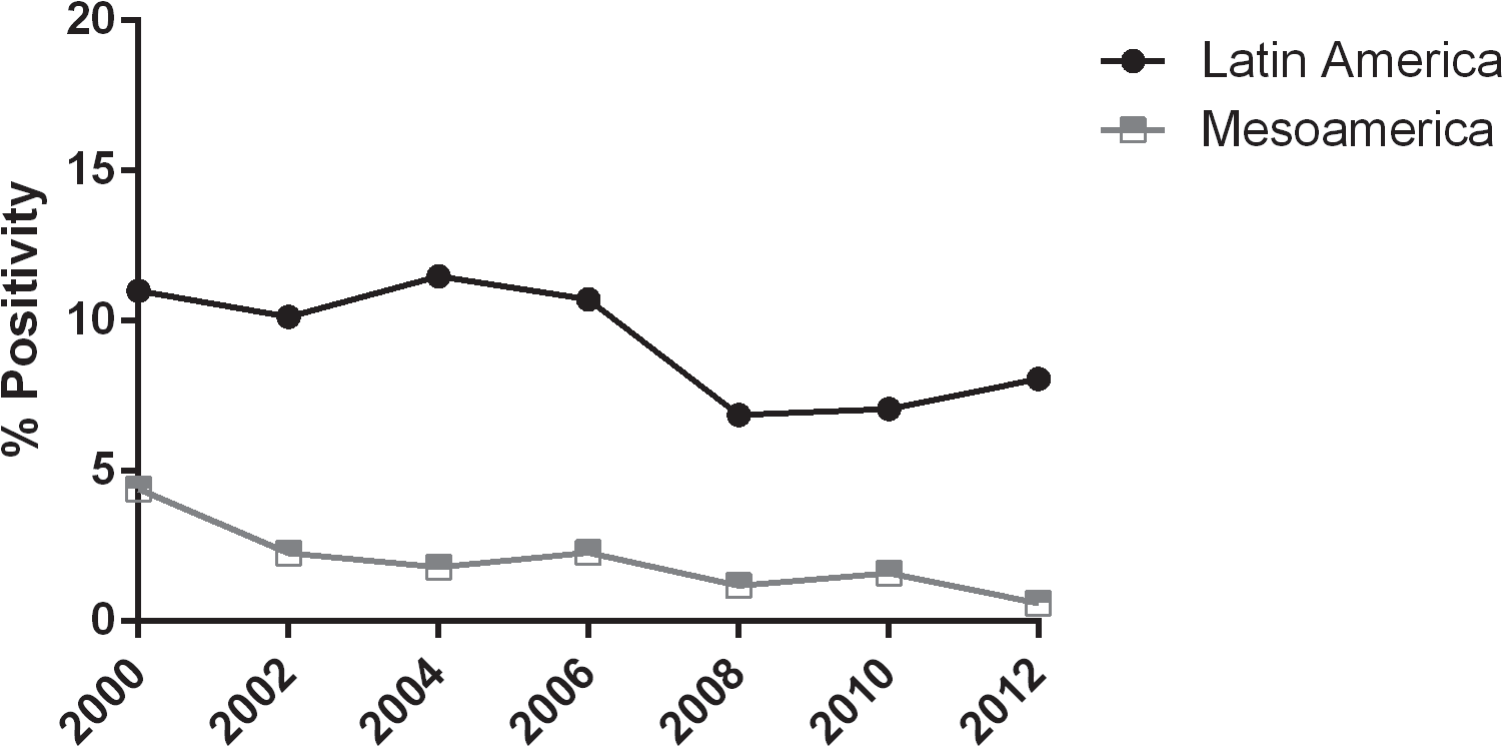
Slide Positivity Rate (SPR) in the last 10 years in Latin America and Mesoamerica.

During the past decade, malaria experienced a remarkable decrease in Mesoamerica, with *P*. *vivax* as the predominant species and only small remaining foci of autochthonous *P*. *falciparum* transmission. In contrast to the rest of the world, in particular SA, both parasite species are still susceptible to chloroquine [13], which is, therefore, routinely administered in Mesoamerican countries for malaria treatment, except for in Panama where *P*. *falciparum* treatment is based on Arthemeter-Lumefantrine [1]. Malaria decreased from ~123,000 cases in 2000 to ~14,798 in 2012, with an epidemic peak occurring in 2005 (Fig 2). Malaria burden has shown a decreasing trend in the Mesoamerican region in the last decade [5]; during this period, the Slide Positivity Rate (SPR) was significantly lower than in the rest of LA (Fig 3).

**Fig 4:**
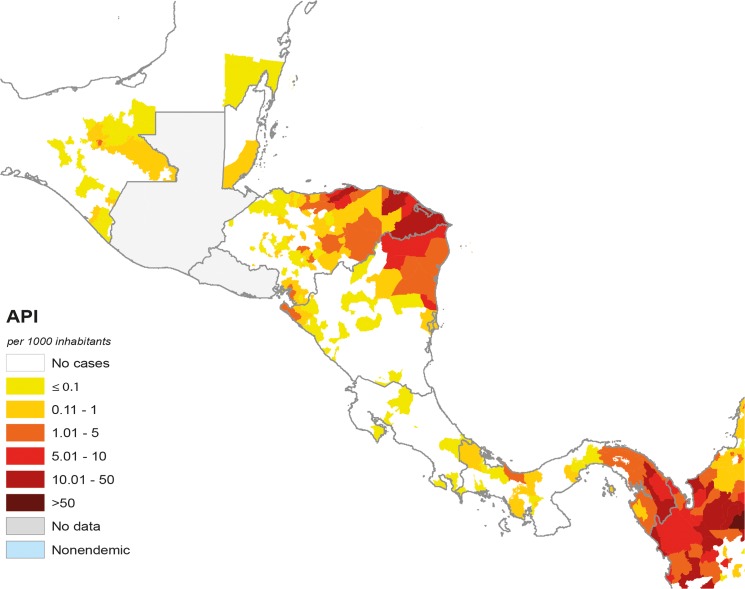
Distribution of malaria in the Mesoamerican region according to the API. Information from the PAHO. *Source*: *PAHO (No data available from Guatemala and El Salvador)*

Comparison of the SPR in America and Mesoamerica shows lower positivity and higher index reduction in the Mesoamerican region in the last decade [5], which correlates with differences in the transmission intensity in both regions. It is remarkable that four of the eight Mesoamerican countries are currently in the pre-elimination phase (Mexico, Belize, Costa Rica, and El Salvador). The latter two reported <20 cases in 2012. Six countries have reached the MDG 6 and RBM goals. Although only Nicaragua and Panama reported an increase in malaria cases from 2011 to 2012, Panama is on track to reach the MDG 6 target in the coming years [1,13] (Fig 4).

La Mosquitia, a tropical rainforest in the northeastern Honduran border with Waspán (Nicaragua), is probably the most endemic Mesoamerican focus. Populated by indigenous communities, and accessed only by boat or airplane, this border region accounted for >70% of *P*. *falciparum* cases in Mesoamerica and ~50% of malaria cases in Nicaragua in 2011 [13]. Guatemala has two transmission pockets: La Gomera (Escuintla department), with 41% of all malaria cases occurring there (100% of *P*. *falciparum* cases in the country), and Gualán (Zacapa department) near the Honduras border [13]. Both of these areas are communities of mestizo population, mainly from rural areas with economic activities based on farming and sugar production [27]. Panama exhibits low malaria endemicity with almost exclusive *P*. *vivax* transmission (99.7%), mainly in El Darien department (a large swath of swampland and forest inhabited by indigenous communities at the Colombian border), with 47% of the Panama malaria cases in 2011 occurring there [13]. Despite these figures, the Mesoamerican malaria map has shrunk recently and the number of cases has decreased to <4% of those in the whole American continent [5].

## Malaria in the Caribbean

Malaria was eliminated in most of the Caribbean islands 45 years ago, except for Hispaniola. Since then, only a few imported cases have been reported in the other Caribbean Islands, although an outbreak with >400 *P*. *falciparum* cases occurred in Jamaica in 2007, and a prompt reaction resolved it over a four month period [28]. Currently, malaria is only endemic in Hispaniola (composed of Haiti and Dominican Republic [DR]) with almost exclusive transmission of *P*. *falciparum*, which remains susceptible to chloroquine, and with *An*. *albimanus* as its main vector. Malaria cases increased from 10,871 in 2001 to 26,375 in 2012, with an epidemic peak in 2010 associated with the earthquake in Haiti when ~86,633 *P*. *falciparum* cases and two *P*. *vivax* were reported [5], although these figures may not be completely reliable due to deficiencies in the malaria information systems. Although a significant reduction was achieved with malaria control interventions to reduce the epidemics associated with the earthquake and the number of autochthonous cases in DR has been decreasing, it may not reach the MDG-6 goal. Malaria in DR has decreased from 3,837 to 952 cases between 2005 and 2012 with a great proportion of cases presumably imported from Haiti [5]. A systematic effort to strengthen the control towards elimination in the island appears timely.

## EMMIE Initiative

Because of the recent reduction trend in malaria transmission (four out of eight countries are in the pre-elimination phase), as well as the relative degree of development of Mesoamerica and DR, a sub-regional initiative aiming at Elimination of Malaria in Mesoamerica and Hispaniola (EMMIE: Eliminación de Malaria en Mesoamerica y la Isla Española) by 2020 has been recently launched led by the GFATM with support from the governments of participant countries. In June 2013, COMISCA subscribed to the “Declaration for the Elimination of Malaria in Mesoamerica and Hispaniola Island by 2020” and instructed its Executive Secretariat (SE-COMISCA) to implement permanent monitoring [29].

In 2014, the GFATM awarded a start-up grant to EMMIE towards malaria elimination in Mesoamerican countries and Hispaniola. Governments of the nine involved countries and of Mexico and Colombia were called into action and focused on the activities listed in Table 1 to be led and monitored by the SE COMISCA, along with important technical advice from other relevant partners such as PAHO, CDC, Clinton Health Access Initiative (CHAI), the Carter Foundation, organized civil society, academia, and other stakeholders. Financial assistance from donor agencies, such as the Bill and Melinda Gates Foundation (BMGF) and others, is being pursued.

**Table 1:** Main activities of SE-COMISCA towards malaria elimination.

Number	Activity
**1**	encourage governments to switch from malaria control to malaria elimination
**2**	strengthen the sub-regional and national political commitment towards malaria elimination
**3**	facilitate joint efforts between participant countries
**4**	accelerate the harmonization of key approaches and strategies
**5**	build upon existing multi-national and cross-border collaboration to support communities most affected by malaria

The Inter-American Development Bank has calculated that eliminating malaria from Mesoamerica in ten years requires US$185 million during the first five years [30], whereas The Carter Center estimates the cost of malaria elimination in Hispaniola at only US$193.9 million [31]. Initial funding of US$10 million granted by the GFATM to EMMIE for a three-year period is therefore a start-up amount to raise awareness and organize the region for elimination as well as to accelerate the control pace, which will require comprehensive education at all levels as well and developing mechanisms to properly evaluate progress. An innovative Results Based Financial (RBF) mechanism known as Cash on Delivery (CoD) has been proposed to reward faster progress in reduction of autochthonous malaria cases reported. Countries would get funds from GFATM only after they reach specific performance targets using the number of autochthonous malaria cases as an indicator. Although governments of participant countries have promised to increase investment in malaria control/elimination activities, there is not, as yet, a clearly defined amount.

In light of the currently available funding, the overall EMMIE goal of eliminating malaria transmission by 2020 appears ambitious as great challenges are to be faced. However, the start-up fund would significantly contribute to stimulate the joint effort of multiple partners to help NMCPs to improve in areas such as: prevention; strengthening of surveillance, treatment coverage, and integrated vector control; as well as to learn to work together within a regional frame. It would help keeping malaria elimination as a key issue in the political regional agenda throughout the next five years and beyond. Moreover, this is a unique opportunity to redirect GFATM resources being allocated for malaria control in several countries of the region, to the EMMIE goal of elimination.

## EMMIE’s Challenges

Despite the favorable malaria epidemiological conditions towards elimination efforts in Mesoamerica and great political support, several technical, administrative, and financial challenges are anticipated for EMMIE implementation.

### Technical Challenges

#### Surveillance and parasitological diagnosis

An effective passive case detection (PCD) system is vital to all phases of elimination and the key challenges are: (i) extending PCD to effectively cover all populations at risk and (ii) shifting to a case-based reporting system that can then facilitate timely case investigation and response (including active case detection [ACD]). However, PCD, the most feasible strategy based on the available infrastructure, experience, and funding, which would be critical in settings with more uncontrolled malaria, such as La Mosquitia in the Honduras-Nicaragua border and La Gomera, Guatemala that would demand further strengthening and quality assurance (QA), may not be sufficient to lead to elimination in the proposed time frame. The diagnosis network needs to be more accessible to the population at risk as well as the proper combination of microscopy and use of rapid diagnostics tests (RDTs). However, in regions where control has been achieved (El Salvador, Costa Rica), there may be a need to rapidly identify positive foci in order to ensure adequate intervention and follow-up, which would require additional strategies to PCD [32]. Despite the additional costs, Reactive Case Detection (RCD) and/or ACD may be appropriate as complementary surveillance strategies with case identification. While PCD focuses on acute cases and contributes to a decrease in disease burden, ACD searches mainly for asymptomatic infections that serve as reservoirs for transmission [33].

Microscopy continues to be the gold standard method both for routine diagnosis and elimination [1], and RDTs are being successfully introduced in remote areas where diagnosis is frequently provided by health workers facing great infrastructural and public services limitations. However, the high occurrence of asymptomatic and submicroscopic infections only detectable by molecular techniques would extend the time and increase the effort and cost of malaria elimination. Significant progress has been made recently in the use of molecular techniques based on detection of parasite nucleic acids [33]. Among them, the loop-mediated isothermal amplification (mLAMP, discussed below) could contribute to improving the detection of asymptomatic carriers in countries with low transmission, such as El Salvador, Costa Rica, and others, as elimination progresses. Although cost-effectiveness analyses of these techniques are needed, it is likely that early detection of asymptomatic carriers and consequent malaria reduction will compensate the higher initial cost. Additionally, communication strategies such as mobile phone use, recently tested in Guatemala in the context of GFATM funded activities, would significantly contribute to accelerating febrile case identification, diagnosis, and prompt treatment, as well as compliance. This represents one of the many examples where private sector (communications) could contribute to the initiative by providing available technologies at accessible costs.

#### Malaria treatment

Chloroquine remains effective for treatment of both *P*. *falciparum* and *P*. *vivax* in most Mesoamerican and Caribbean regions despite the early appearance of *P*. *falciparum* resistance to chloroquine in SA several decades ago [14,34], and more recent reports of *P*. *vivax* resistance as well [35]. Although chloroquine is still the first line treatment in the whole sub-region, except in Panama where Arthemeter-Lumefantrine is used [1], active chloroquine resistance surveillance becomes critical, especially due to the proximity of Mesoamerica with Colombia and the increasing mobility through the region. The policy in most Mesoamerican countries is radical treatment for *P*. *vivax* with chloroquine for three days and primaquine for 14 days, except in Nicaragua, Panama, and Costa Rica where primaquine treatment is given for seven days only [1]. As *P*. *vivax* is the predominant *Plasmodium* species in Mesoamerica, adherence to the longer primaquine treatment is essential to prevent relapses and to accelerate elimination [36]. According to available reports, the most efficient treatment regimen is the use of a schizonticide drug and primaquine for 14 days (0.15 mg/kg/day) instead of shorter five and seven days regimens [37]. A harmonized treatment schedule should be pursued by EMMIE, as well as efforts to improve treatment compliance. Although directly observed treatment (DOT) with primaquine and patient follow-up to ensure adherence in practice are not easy to implement, depending on the setting, training of community workers and use of mobile phones could be attempted as *P*. *vivax* relapses due to incomplete treatments would seriously affect progress towards elimination. Tafenoquine, a new 8-aminoquinoline drug derived from primaquine (PQ) with longer half-life and potential anti-relapse effect, may become available in the next few years [38]. Tafenoquine is administered as a significantly shorter treatment, facilitating *P*. *vivax* treatment including DOT.

#### Vector control

Vectors present in Mesoamerica are *Anopheles albimanus*, *An*. *vestitipennis*, and, to a lesser extent, *An*. *punctimacula s*.*l*, *An*. *pseudopunctipennis s*.*l*.*i*., and *An*. *darlingi*. *An*. *albimanus* is the most abundant and widely distributed species [39], and, despite its more zoophilic behavior, it is able to maintain human malaria transmission in the region. *An*. *vestitipennis* is considered a secondary species of major importance, although in Belize it is a primary vector, carrying both *P*. *falciparum* and *P*. *vivax* [40]. It has also been incriminated in malaria outbreaks in the Caribbean islands [41]. The role of *An*. *punctimacula s*.*l*. is controversial due to its unresolved taxonomic status.

Unfortunately, human exposure frequently occurs outside the range of effectiveness of the current indoor control measures (indoor residual spraying [IRS] and insecticide-treated nets [ITNs]). Vector control in Mesoamerica is based on the use of long-lasting ITNs and IRS, together with elimination or intervention of mosquito breeding sites [42,43].

One of the main threats to vector control in this region is insecticide resistance shown by *An*. *albimanus* and other local species [44–46]. Recently, a *Kdr* mutation, which confers resistance to DDT and pyrethroids, was described in samples from Mexico, Nicaragua, and Costa Rica [47]. Therefore, surveillance on insecticide resistance must be strengthened.

### Administrative and Financial Challenges

For EMMIE to succeed, some administrative changes are essential (Table 2) regarding protocols harmonization, strengthening and retraining of personnel, as well as regional policies. As the initial US$10M granted to EMMIE by GFATM would be largely insufficient to achieve malaria elimination in Mesoamerica [29,31], this funding is only conceived to stimulate countries to shift from control to elimination. Several countries (Guatemala, Honduras, Nicaragua, Dominican Republic, and Haiti) have received GFATM grants that could be harmonized with the EMMIE strategies. However, a significant injection of funds from other donors is required, as well as coordination of the growing number of EMMIE participants to reduce effort duplication and reduce costs. Another issue of concern is that success and CoD are currently envisaged based on a reduction of autochthonous cases reported, which may lead to the interpretation that an increase in detected cases due to better surveillance represents a failure in control activities, whereas a decrease shows success. CoD may, therefore, work better once diagnosis/detection methods have improved in the region. An initial investment to ensure community participation may prove highly efficient and cost-beneficial, in particular if it is supported by modern technology, such as mobile phones and others, to improve timely treatment.

**Table 2:** Administrative challenges faced by EMMIE.

Number	Administrative Challenge
1	A change in paradigms and re-training of technical personnel, as well as reinforcement of the programs’ manpower is required for shifting from control to elimination
2	Retraining must include harmonization of the NMCP protocols and malaria should be faced as a regional instead of a per-country issue
3	Cross-border malaria has to be taken into account and treated independently regardless of where cases originated
4	Diagnosis network needs to be strengthened with quality assurance, including ACD whenever needed and convenient
5	The CoD funding mechanism may be troublesome as it implies that countries get rewarded based on reduction of the number of autochthonous malaria cases; the number of malaria cases is highly likely to increase as diagnose improves
6	Case recording may not be reliable enough if a robust information system is not in place

## Knowledge Gaps and Research Agenda for the EMMIE

Multiple knowledge gaps are revealed when shifting from malaria control to elimination that underscores the need for a comprehensive research agenda. The Malaria Eradication Research Agenda (MalERA) [48,49] was provided by the RBM Global Malaria Action Plan and the Malaria Elimination Group, under the sponsorship process. At the regional level, PAHO has devoted considerable effort to defining a regional malaria research agenda for the continent [50] considering current malaria elimination initiatives in Mesoamerica. However, research needs would change locally and would require significant involvement of the NMCPs, local academia, as well as external technical support. Knowledge gaps range from social, economic, and anthropological to more biological and basic knowledge questions, from identifying the means to communicate and involve indigenous communities, as well as survey the sensitivity of parasite and mosquito populations, to current anti-malarials and insecticides, to prevalence of genetic traits of the communities that may influence malaria elimination. An assessment of G6PD deficiency in the EMMIE communities would be essential as more people are likely to be treated in an elimination program.

Diagnosis is probably the most critical issue for defining elimination strategies. Great efforts should be invested in defining how best to use PCD in areas of greater transmission intensity, as well as where and how to approach the need for ACD in the search for asymptomatic cases. The usual low parasitemia present in settings of low malaria transmission will be better detected by more sensitive diagnostic tools. However, these require sophisticated equipment and well-trained personnel, which restrict them to research purposes only [51–53]. The recent availability of new molecular techniques holds great promise if developed and tested for field use [33,54]. Malaria LAMP, with equivalent performance to polymerase chain reaction (PCR), requires shorter time, less equipment, and easier reagent transportation and storage [55]. Sensitive field-deployable diagnostic methods, although not essential for implementation of surveillance and response activities in malaria elimination campaigns, will aid in detecting low levels of parasitemia in asymptomatic individuals who may still carry gametocytes and, therefore, contribute to transmission to mosquitoes [56].

Another critical component of malaria elimination is vector control, particularly evaluation of the efficacy of presently used strategies. Human exposure outdoors (not blocked by IRS and ITNs use) should be determined, as well as definition of the primary and secondary mosquito species.

A better understanding of indigenous communities that are frequently located in malaria hot spots is required, together with improved management of mobile communities. As most cases come from transmission pockets in remote areas with limited access to health services, new strategies for screening, treatment, and vector control for these populations should be developed.

Capacity building and education of communities, health care providers, and decision makers should also be on the research agenda, as well as parasite and host biological factors. In addition, for malaria elimination approaches to succeed, unsatisfied basic needs in the endemic communities need to be considered, including poverty in Haiti (low income country). Panama, Costa Rica, and Dominican Republic are upper middle-income countries [57].

As research will provide important answers regarding elimination tools, academic institutions will play an important role in EMMIE’s activities. Mesoamerican countries and Hispaniola are included in the geographical area covered by the Latin American Center for Malaria Research (CLAIM), one of the International Centers of Excellence for Malaria Research (ICEMRs), sponsored by the National Institute of Allergy and Infectious Diseases of the National Institutes of Health (NIAID/NIH), United States of America, to generate evidence to support further malaria elimination strategies. CLAIM, based on its research and databases (Table 3), will provide technical and scientific support to the NMCPs in the region (including Guatemala, Panamá, Honduras, and, possibly, future activities in Hispaniola) to strengthen malaria control activities and accelerate elimination.

**Table 3:** CLAIM’s accomplishments.

Research areas	1) epidemiology of malaria transmission in low to moderate transmission settings in LA 2) vector biology and integrated vector management for malaria control 3) immune-pathogenesis of malaria in the region
Data management	Establishment of a data management core to allow interaction and data sharing between all associated research groups and a dynamic research-training component to leverage research capacity of science and malaria workers
Training and courses offered	Three workshops/training courses were offered by CLAIM in 2014 together with the Colombian Ministry of Health and the Presidential Agency for International Cooperation (APC-Colombia) on malaria diagnosis for elimination, the use of Geographic Information Systems for the design of malaria risk maps and vector taxonomy and control

The Barcelona Institute for Global Health (ISGlobal), an EMMIE affiliate, led an international course on “Eliminación de la malaria en Mesoamérica y La Española” held in San Salvador in agreement with COMISCA and support from BMGF. Both academic partners are focusing efforts on strengthening the regional scientific capacities and critical mass. An active involvement of the local universities and research institutions will contribute to strengthening the research capacity in the region; Reference Centers should be established in different countries with support of local universities, research centers, and collaborators.

Other agencies and foundations have contributed to the EMMIE initiative including CHAI, BMGF, the CDC, the Carter Center, the Inter-American Development Bank, the Foundation for Innovative New Diagnostics (FIND), and the MoHs from Colombia and Mexico; these two countries provide support and advice based on their own experience in malaria control.

Even if challenges and knowledge gaps are faced properly and EMMIE progresses successfully, the budget specifically allocated, funding that GFTAM contributes through other grants, as well as the potential increase from countries to their NMCPs, appear largely insufficient. Malaria elimination is only accepted once WHO certifies a region had zero malaria cases for at least three consecutive years. Other examples from the continent and abroad show that initially successful projects can fail when initial conditions change. During the first half of the past century in Venezuela, malaria was the first infectious disease killer [58] by the mid-1950s, at the beginning of the Global Malaria Eradication Program (GMEP), but it had eliminated malaria from half of its territory to reach 77% malaria-free by 1971. Malaria started increasing again in the early 1980s due to agricultural laborers migrating through malaria-free areas with immunologically-susceptible populations and rapid proliferation of hot spots [59]. The great success based on systematic and integrative infection and DDT vector control could not be maintained and Venezuela is one of the few countries in the continent with recent malaria increase, reaching 76,621 cases in 2013, second after Brazil [59]. Another example is Sri Lanka where malaria caused great devastation during the first part of last century with 5.5 million cases by 1935. Sri Lanka, as Venezuela, had established a very successful malaria control program before the GMEP, but joined it in 1958. A massive incidence decline followed and only 17 cases where recorded in 1963 [3,6,7]. Then, control activities were relaxed, financial support was reduced, and rainfall increased [60,61], leading to a massive resurgence, with an estimated 1.5 million cases in 1967–1968 [62,63]. Major epidemics occurred in the 1980s and early 1990s [64].

From 1999 onwards, Sri Lanka achieved major reductions in incidence and may now be considered a controlled, low-endemic country [65] aiming to interrupt indigenous malaria transmission by the end of 2014 [66,67].

## Conclusions

The significant recent progress in Mesoamerica as well as the strengthening of malaria control measures in Hispaniola represent a great opportunity for malaria elimination in the region. The EMMIE initiative’s goal is to bring malaria transmission to zero by 2020. Although an initial seed grant of US$10M to incentivize the work during 2014–2107 is greatly insufficient, it is generating great enthusiasm among governments in the region, which have promised additional funding. However, substantial additional funding is required from donors to secure the estimated US$180 million needed. A broad call to bring together numerous stakeholders is ongoing and a number of external partners have already joined the initiative. Great challenges and knowledge gaps are being faced, but valuable joint work by academic partners resulted in considerable progress in 2014.

**Box 1. Key learning points.**

- The EMMIE initiative has stimulated the interest of technical and funding agencies, NGOs, academia, and other stakeholders. The success of this endeavor is highly dependent on the concerted action and collaboration of interested parties.
- Participating countries shall sustain and increase the actual political will, translating it into concrete sub-regional and national actions to achieve and sustain malaria elimination.
- Various research activities are required to better understand the impact of different strategies for malaria elimination.
- The success of the EMMIE program will accelerate malaria elimination in specific regions of SA while serving as a basis for malaria pre-elimination in other regions of the world.

**Box 2. Key papers in the field.**

- World Health Organization (2013) World Malaria Report 2013
- Feachem RG, Phillips AA, Hwang J, Cotter C, Wielgosz B, Greenwood B, et al. (2010) Shrinking the malaria map: progress and prospects. The Lancet 376: 1566–1578.
- Alonso PL, Brown G, Arevalo-Herrera M, Binka F, Chitnis C, Collins F, et al. (2011) A research agenda to underpin malaria eradication. PLoS Med 8: e1000406.
- Herrera S, Quinones ML, Quintero JP, Corredor V, Fuller DO, Mateus JC, et al. (2012) Prospects for malaria elimination in non-Amazonian regions of Latin America. Acta Trop 121: 315–323.
- Hopkins H, González IJ, Polley SD, Angutoko P, Ategeka J, Asiimwe C, et al. (2013) Highly Sensitive Detection of Malaria Parasitemia in a Malaria-Endemic Setting: Performance of a New Loop-Mediated Isothermal Amplification Kit in a Remote Clinic in Uganda. Journal of Infectious Diseases.
